# Reporting and appraising the context, process and impact of PPI on contributors, researchers and the trial during a randomised controlled trial - the 3D study

**DOI:** 10.1186/s40900-018-0098-y

**Published:** 2018-05-14

**Authors:** Cindy Mann, Simon Chilcott, Katrina Plumb, Edmund Brooks, Mei-See Man

**Affiliations:** 1 Centre for Academic Primary Care, University of Bristol, Bristol, UK; 2 PIP-CaRE group, University of Bristol, Bristol, UK

**Keywords:** Patient public involvement, Qualitative, Randomised controlled trial

## Abstract

**Plain English summary:**

Including patient and public involvement (PPI) in health research is thought to improve research but it is hard to be clear exactly how it helps. This is because PPI takes many forms, is sometimes only token and is not always reported clearly. This makes it difficult to combine the evidence so that clear conclusions can be reached about the ingredients of successful PPI and what PPI achieves. Previous research that has tried to combine the evidence has led to several guidelines for researchers to use in setting up and reporting PPI.

This paper was written jointly by researchers and PPI contributors as a reflection on our experiences. The aim was to add to the evidence, by giving detail about the use of PPI in a large randomised controlled trial and the effect it had. We were guided by published PPI reporting guidelines. The effects on the trial are shown in a table of changes made because of suggestions from the PPI group. A survey was used to ask PPI contributors and researchers about their experience and effects they had noticed. Three themes were noted: impact on the trial, the effect of involvement on individual researchers and group members, and group environment. The PPI work affected the trial in many ways, including changes to documents used in the trial and advice on qualitative data collection methods and analysis. Individuals reported positive effects, including enjoying being in the group, gaining confidence, and learning how to share views.

**Abstract:**

****Background**:**

Patient and public involvement (PPI) is believed to enhance health care delivery research, and is widely required in research proposals. Detailed, standardised reporting of PPI is needed so that strategies to implement more than token PPI that achieves impact can be identified, properly evaluated and reproduced. Impact includes effects on the research, PPI contributors and researchers. Using contributor and researcher perspectives and drawing on published guidelines for reporting PPI, we aimed to reflect on our experience and contribute evidence relevant to two important questions: ‘What difference does PPI make?’ and ‘What’s the best way to do it?’

****Methods**:**

Fourteen people living with multiple long-term conditions (multimorbidity) were PPI contributors to a randomised controlled trial to improve care for people with multimorbidity. Meetings took place approximately four times a year throughout the trial, beginning at grant application stage. Meeting notes were recorded and a log of PPI involvement was kept. At the end of the trial, seven PPI contributors and four researchers completed free-text questionnaires about their experience of PPI involvement and their perception of PPI impact. The responses were analysed thematically by two PPI contributors and one researcher. The PPI group proposed writing this report, which was co-authored by three PPI contributors and two researchers.

****Results**:**

Meeting attendance averaged nine PPI contributors and three to four researchers. The involvement log and meeting notes recorded a wide range of activities and impact including changes to participant documentation, advice on qualitative data collection, contribution to data analysis and dissemination advice. Three themes were identified from the questionnaires: impact on the study, including keeping the research grounded in patient experience; impact on individuals, including learning from group diversity and feeling valued; and an environment that facilitated participation. The size of the group influenced impact. Researchers and PPI contributors described a rewarding interaction that benefitted them and the research.

****Conclusions**:**

PPI was wide-ranging and had impact on the trial, contributors and researchers. The group environment facilitated involvement. Feedback and group interactions benefitted individuals. The insights gained from this study will postitively influence the researchers’ and contributors’ future involvement with PPI.

## Background

It is generally accepted that patient and public involvement (PPI) enhances the value of health care delivery research by making it more relevant to the targeted patient group [1]. Consequently, PPI is now required in all health care research undertaken for funders such as the National Institute of Health Research (NIHR) in the UK [2]. However, this may lead to tokenism [3–5] and several reviews of PPI have concluded that evidence of impact is weak [5–8]. A better evidence base is needed to substantiate claims of benefit and to evaluate impact and cost-effectiveness [9]. This would also facilitate learning about how to achieve effective PPI and what key factors operate when it does have impact [10–12].

Achieving a robust evidence base requires more comprehensive, structured reporting of PPI in individual studies [2, 3, 6, 13, 14]. Many suggestions have been made about what should be included in planning and reporting PPI [3, 5, 6, 12–19] and some detailed tools have recently become available [12, 17, 20]. Some argue that the complexity of PPI makes it difficult to assess its impact on research beyond documenting contributions [21]. Others have noted that the impact on individuals, both contributors and researchers, should not be overlooked [13]. Attempts have been made to categorise PPI activity to aid reporting [14] and to design a structured set of questions to plan and prompt reflection on PPI [18]. Some consensus has developed about what leads to successful PPI in a research study, including planning how to incorporate PPI, initiating PPI from the outset and good relationships between researchers and PPI contributors [3, 7, 14, 15]. Reports of PPI should include contextual details, because the mechanisms for successful PPI will vary with the type of study, PPI aims, resources available and what values are held by researchers, particularly the Chief Investigator, regarding PPI [3, 5, 8, 10, 13].

This paper describes the PPI processes within a randomised controlled trial (RCT) setting and analyses the perceptions of both PPI contributors and researchers regarding the process and impact of the PPI and the challenges they experienced. This addresses a relative gap in the literature as reports of PPI from both researcher and contributor prespectives are under-represented, as are reports of PPI in RCTs [15]. Following Crocker [21] and Dudley [15], we have used the term ‘PPI contributor’ to refer to the members of the PPI group, as they were not co-applicants in the research proposal or co-researchers in carrying out the research. Instead they were consulted and actively contributed their ideas to help shape how the research was carried out.

The paper was initiated by the PPI group towards the end of the trial, who wished to record their contribution to the trial and their experience. We aimed to create a PPI report that included recommended detail on context, process and impact to add to the evidence base and which would facilitate evidence synthesis by conforming to published reporting guidelines as far as possible [3, 12, 13, 17, 20]. Thus, the main aim of this paper is to contribute evidence towards understanding how and in what circumstances PPI makes a difference. We also aimed to provide an example of how researchers and contributors with little experience of PPI succeeded in overcoming tokenism, achieved meaningful collaboration and experienced individual impact. We hope that this will be encouraging and informative to other researchers in a similar position.

### Description of trial

The PPI group contributed to a large cluster RCT (The 3D Study; ISRCTN06180958) that took place in 33 general practices in three areas of the UK. The aim of the RCT was to improve the care of people with a wide range of multiple long-term conditions (multimorbidity) by addressing patient-reported generic deficiencies in their care and issues that negatively impacted their quality of life [22]. The intervention aimed to enhance continuity of care and establish a more patient-centred approach to reviewing patients with multimorbidity by eliciting patients’ own agendas, reviewing all conditions together and agreeing shared action plans to address issues. Full details are available in the protocol paper [22]. The trial design included an economic evaluation to examine cost-effectiveness and a process evaluation [23] to explore participants’ response and how and why the intervention was implemented or not. The intervention ran over 15 months and involved 1546 patients, 797 of whom were in the 16 intervention group practices. The trial funding began in March 2014 and concluded in September 2017. The funding proposal took over a year to develop prior to the start of the trial and included PPI funding of £13,612 plus 5% FTE of one researcher to co-ordinate the PPI, representing approx. 1.1% of the total cost.

### Aim of PPI

The aim of the PPI was to ensure maximum relevance of the trial intervention to people with multimorbidity, to enhance response to recruitment and follow-up, to help interpret results and to gain assistance with dissemination to interested groups and individuals. Plans for PPI described in the proposal specified meeting 4 times a year to advise on patient information leaflets, questionnaire design, ethical issues, recruitment approaches, dissemination of results, and impact. We also stated the intention to identify two members of the group willing to sit on the Trial Steering Committee and the Trial Advisory Group and another member to contribute to the qualitative data analysis. The PPI group was named the Patient Involvement in Primary Care Research (PIP-CaRe) group to give it an identity and to reflect a wider aim of it being a resource that researchers developing new primary care research ideas could use.

## Methods

During proposal development, PPI co-ordination and facilitation was delegated to a named co-applicant (CM). An open meeting to discuss the research proposal was advertised with relevant patient groups and local PPI facilitators. Four people responded to the first invitation. There were no formal selection procedures beyond the criterion of living with multimorbidity as a patient or carer. Recruitment continued until, in December 2013 just prior to the start of the research programme, an introductory meeting was set up with fourteen PPI contributors. Two had prior experience of PPI and two were co-ordinators of voluntary patient groups. The PPI co-ordinator had minimal previous exposure to PPI and no previous experience of facilitating PPI groups but had prior training in group facilitation. After becoming co-ordinator she attended two one-day courses on PPI in research that drew attention to important facilitation considerations such as the group’s needs and different motivations.

Eleven PPI contributors remained in the PIP-CaRe group after a year, of whom 3 were male. The age range was approximately 45–85 and contributors had a variety of long-term conditions. They all continued to attend meetings throughout the trial, averaging nine PPI contributors at every meeting. Following the introductory meeting, the group met four times in 2014, three times in 2015, and four times in 2016 and 2017. Between meetings, PIP-CaRe members were often asked for their opinions by email. They were compensated for their time at the rate of £12 per hour for meetings, preparation work, and for additional work or reading undertaken between meetings. These payments were made in vouchers and payments for travel expenses were in cash. Meetings were board-room style on university premises with lunch provided. Diet, mobility, sight and hearing needs were accommodated where possible with alternative food options, access on the level or by lift, large font for meeting papers and rooms with good acoustics. Two other members of the lead site research team also attended meetings regularly; the trial manager (MM) and the main trial researcher. The Chief Investigator of the trial was supportive of PPI and attended approximately one third of meetings. Occasionally other researchers attended for a specific purpose, such as development of a new patient-reported measure used in the trial. In the first year, the PPI co-ordinator provided half a day’s training in trial methodology to PIP-CaRe members. They were also encouraged to attend free training at a local PPI hub. Nine members attended the trial methods training and five attended the local PPI training. One year into the trial, one member left the group following unresolved conflict with the PPI co-ordinator when differing participation requirements within the group became incompatible. Following this, the functioning of PIP-CaRe was appraised and members were asked by questionnaire about their needs in attending the group. This resulted in some adjustments to the physical environment. The group discussed whether they were happy with how PIP-CaRe was chaired and facilitated and chose not to make any changes.

### Data

Throughout the trial, a log was kept of all PPI activity and contributions. Detailed notes of PIP-CaRe meetings were written up by the PPI co-ordinator immediately after the meeting and circulated to all attending researchers to check accuracy. Within a week of the meeting, the notes were sent to the whole group, who were also asked to amend anything they felt to be inaccurate. Those who had not attended were also sent a copy so that they remained informed.

Towards the end of the 3D Study both PPI contributors and researchers were invited to comment on their experience of working with the PPI group. A questionnaire, developed with input from PIP-CaRe, was provided to group members and to researchers. The purpose was to prompt reflection on both process and impact and enable all involved to relate their experiences in general terms, whilst providing specific examples where they wished. Both questionnaires are shown in Table 1. The PPI co-ordinator did not complete the questionnaire but her reflections are included in the discussion.

**Table 1:** PPI contributor and researcher questionnaires

PPI contributor questions	Researcher questions
Why do you think it is important that the group was involved?	Why do/did you think it is important that the group was involved?
	What do/did you see as the group’s purpose in the study?
What do you think has been the most important contribution made by: a. The group? b. By you?	What do you think has been the most important contribution made by the group?
What other contributions have been made?	In your opinion, what other contributions have been made?
What has given you the greatest satisfaction in working with the 3D study?	What has given you the greatest satisfaction in working with the group?
What barriers and facilitators have there been to you taking part in the PIP-CaRe group?	What (if anything) has been difficult or challenging in working with the group? a. For the trial? b. Personally?
	Where have you experienced unexpected benefits from working with the group? a. For the trial? b. Personally?
What lessons do you think could be learned from the experience? a. Personally? b. By researchers?	What lessons do you think could be learned from the experience? a. Personally? b. By researchers in general?
Anything else you would like to add?	Anything else you would like to add?

### Analysis

The meeting notes were scrutinised for examples of impact and used to check the accuracy of the activity log. The questionnaire answers were first collated question by question into PPI contributor responses and researcher responses and drafted into a narrative by two PPI contributors (SC and KP). The narrative was then discussed in the whole group who made some additional points, particularly about the PPI perspective on quality of life. The collated comments were thematically analysed using codes that arose from the data. The codes were grouped into themes which were agreed between CM, SC and KP.

## Results

First, we present the PPI activities and demonstrable impact based on the meeting notes and activity log that were kept throughout the trial, followed by the findings from the questionnaires addressing perceptions of PPI contributors and researchers.

The activities the PPI contributors were involved in were very wide-ranging and included all those specified in the funding proposal as well as others. The main activities that had impact on the trial conduct are shown in Table 2. A full list appears in Appendix 1.

**Table 2:** PPI activity log

Activity	Demonstrable impact
Considered the research proposal and its relevance to the priorities of the targeted group of patients	Could state in the funding proposal that the intervention addressed the priorities of patients with multimorbidity.
Commented on study design and advised on consent procedure and patient response	After the pilot study, the trial management group decided that a specific decline form would not be included in the invitation pack for the trial. However, the PPI group felt that there should still be a means to actively decline so that patients were not sent reminders. We agreed that participants could return a blank questionnaire as a means of declining and changed the invitation letters accordingly. Only those who did not send any response to the invitation were reminded. The group later highlighted concerns that patients might have about their questionnaire responses being seen by their health care team. As a result, a statement was added to the front page of follow-up questionnaires that patients’ answers would not be shared with their surgery
Gave feedback on recruitment documents for patients including information sheets, consent forms and invitation letters to patients	Information sheets were changed from a two-column format to one and the language was simplified and clarified.
Gave feedback on patient questionnaires	Changes made to the formatting to make them easier to read for partially-sighted people and the layout clearer. Changes to the first page to emphasise appreciation of patients taking time to complete them. Some things they would have liked to change, e.g. wording of questions, were not possible due to the use of validated measures.
Contributed to developing a new measure of treatment burden	The questions in the measure were shaped by PPI contributors’ responses to an existing measure and the new measure was tested on them and refined before being piloted
Took part in a pilot focus group to help develop the focus group schedule for patients participating in the process evaluation	The order of the focus group schedule was changed following feedback to make it flow better and the questions were made more open.
Contributed to development of training for clinicians taking part in the trial	Provided comments on their care and what was important to them in receiving care for multimorbidity to use as quotes in the training slides. One PPI contributor was video-recorded in a role play consultation with the CI for use in training. However, this was not eventually included. Emphasised the need for the training to put the patient’s perspective more strongly which was reflected in revisions to the training made following the pilot phase.
Advised on content and format of the study webpage and contributed to content	Wording was changed from managing patients with multimorbidity to treating patients with multimorbidity. A picture was changed to show a female GP with a male patient. Suggestions for links to other resources were incorporated. A video was recorded of several PPI contributors discussing the 3D study of which an extract was uploaded to the website.
Advising on qualitative interview schedules and on what to look for in observation of consultations that would indicate patient-centredness from their perspective	The interview schedules and observation checklists were revised to incorporate their comments
Two PPI contributors assisted with qualitative data analysis as second coders and the group commented on selected transcripts and recordings	The PPI contributors coding affirmed that of the process evaluation researcher and the PPI group’s comments helped with judging the patient-centredness of recorded intervention consultations
Providing feedback on a planned conference presentation	Changes were made to the presentation that reflected PPI contributor comments and made it easier to follow by changing the order of the slides
Advised on content and format of the patient newsletter	Suggestions regarding pictures and content were incorporated e.g. changing the wording from management to treating and the text in the dark boxes was changed to a colour better for visually impaired people
Dissemination	The group suggested means of disseminating to community and patient groups that were incorporated into the dissemination plan
Publishing their experience of PPI in the 3D study	Several PPI contributors initiated the idea of writing a paper on the PPI in the 3D study which has led to this paper. Three of them (SC, KP, EB) have been actively involved in drafting it.

Seven PPI contributors and four researchers completed the questionnaires. The findings are grouped into research impact, personal impact and environment. In attributing quotes, researchers are designated by the numbers R1–4 and PPI contributors by the numbers P1–7.

### Research impact

PPI contributors and researchers agreed that PPI contributors had played an essential role in the 3D Study. They had bridged the divide between the academic and “real world” by presenting their real-life experiences to provide researchers with a “sounding board” and a “reality check”. They could maintain focus on how participants might be experiencing the intervention and provide insight into their likely responses.

*“It was vital that you had a group of people who could give you a wide cross section of views and experiences on how we have been treated with regard to multiple health issues in the real world …. to keep you grounded.” [P4]*

*The group was needed “to provide feedback and ‘reality-testing’ on our ideas, plans and research tools; to contribute a range of different perspectives.” [R2]*

Nevertheless, one PIP-CaRe member perceived that the research team was sometimes insufficiently sensitive to patients’ perspectives.

*“on occasion researchers paid not enough respect to the ‘subjects’ selected for the research…I sometimes felt that the subjects were taken for granted.” [P2]*

Many PPI contributors highlighted their contribution in making written materials more accessible to the recruited patients.

*“We have achieved paperwork that makes sense to people and is useful to them.” [P3]*

But, one researcher, who was developing a new measure for use in the trial, thought high levels of literacy within the PIP-CaRe group, when compared with the general population, may have reduced the generalisability of their advice.

*“In terms of developing the questionnaire, with hindsight it may have been more appropriate to conduct the cognitive interviews with patients who were more similar to patients taking part in the main trial and to the general population … members of the PIP-CaRe group tended to have high levels of literacy.” [R1].*

Group members claimed they had influenced how data were collected and interpreted.

*“We have suggested reasons why different things like the death-rate in the project might have happened.” [P3]*

Their experiences of dealing with social support networks and the wider community were also useful.

*“I think that the unexpected benefits have come from finding out about other organisations and groups that the members have been involved in. Some of the group are very active in community projects and have connections with local organisations.” [R3]*

Researchers commented on the genuine interest and engagement from PIP-CaRe members that led to meaningful discussion and challenge. One researcher concluded that the size and mix of the PPI group enabled them to challenge, leading to greater impact in an environment where people could be heard.

*“The PIP-CaRe group have the confidence to be challenging, to ask difficult questions of the research team…. because it is a larger group, which has some strong personalities and this gives it more confidence and clout than just having two PPI reps on the project group.” [R2]*

Group members perceived that their ideas and opinions had a direct and positive impact on the research.

*“I’m sure the researchers have been challenged and occasionally surprised by the opinions of the group.” [P1]*

Both researchers and PPI contributors valued the group’s diverse experience and recognised that everyone had something to contribute and should be listened to even when opinions differed.

*“…all the PIP-CaRe group make different sorts of contributions, at different levels and in different ways. So there is no best way to do it, we just need to listen to everyone and hear what they are saying.” [P5]*

*“I believe that the PIP-CaRe group contains a varied group of people with diverse backgrounds, opinions and life experiences. We have not always agreed with one another, but we have always been encouraged to speak and we’ve been heard.” [P1]*

Sometimes researchers struggled with the group’s lack of understanding and/or acceptance of the boundaries and constraints of the research and one researcher felt frustrated when she could not incorporate a good idea because of those constraints

*“I found it frustrating and felt it a weakness on my part that we couldn’t make all the changes the group suggested e.g. changing validated questionnaires.” [R3]*

### Personal impact

Both researchers and contributors found that communication could be complicated by the diversity of the group. However, it could lead to individual learning. One researcher had learnt to take time to understand what PPI contributors were saying and to communicate more clearly herself.

*“I think that I have learned to have more patience to allow PPI members time to explain themselves. I think I have also had to learn how to explain myself clearly.” [R3]*

Several PPI contributors realised the importance of listening to others and understanding the different ways in which they expressed themselves.

*“I need to be a better listener. I am used to meetings …… where the participants are used to focus clarity, external constraints and the need to quickly reach a resolution. I had to step back from that environment and listen to people who expressed their views in a more indirect and personal way.” [P6]*

When asked about their experience of being in the group, PPI contributors described being involved, enthusiastic, listened to and valued. The benefits and enjoyment of working with other group members who brought different experiences and ideas to the discussion could be personally enriching.

*“I thoroughly enjoyed contributing to the group and the ideas that drove the project forward.” [P6]*

*“Personally I feel I have gained experience of medical conditions. I have enjoyed meeting other members, and hearing the views of others.” [P2]*

Many placed personal interactions with researchers and PPI contributors among the most satisfying and enjoyable aspects of working on the study. Researchers gained from what the PPI contributors were willing to share about their lives and PPI contributors stated they had gained knowledge about research.

*“I have particularly enjoyed learning about the process involved in carrying out a research project, and the interactions with the research team and the other members of the PIP-CaRe group.” [P1]*

*“[It has given me] greater understanding of the patients involved in the trial and all the other factors that impact their lives daily yet most remain so positive (both PPI and trial patients)!” [R4]*

The genuine engagement of the the PPI contributors made the research seem worthwhile and was very rewarding for researchers

*“The greatest satisfaction has been developing a rapport and relationship with individuals and the group. It reminds me that the ultimate aim is to help patients’ wellbeing.” [R3]*

*“Seeing how much they have bought into the trial and how much they view it as theirs – with pride.” [R4]*

From the perspective of the PPI contributors, the sense that they had the ability to make a difference was the main source of satisfaction

*“Seeing suggestions or comments I have made used and that you have seen what I have to say as useful to others.” [P4]*

There was also some evidence that PPI contributors had gained confidence as a consequence of their involvement.

*“I feel I have gained more confidence in meeting members of the medical profession- might find discussion with them easier.” [P2]*

*“I think I have had the knowledge that it is not necessary to have higher education to be able to speak coherently and cogently, reinforced.” [P3]*

### Environment

The group appreciated the researchers’ efforts in trying to meet PPI contributors’ needs regarding travel, timing of meetings, room access, diet, sight and hearing. However, some PPI contributors experienced difficulties attending meetings due to illness, treatment demands, employment or other commitments.

PPI contributors were unaware of any barriers to participation and most comments emphasised the experience in a positive light. Overall, the group seemed able to talk freely, and equally and were receptive to the views of others.

*“I did not feel there were any barriers to participation, which was always welcomed by the researchers.” [P2]*

## Discussion

The process that influenced PPI contributors’ impact on the trial included appointing a designated co-ordinator, inclusive recruitment to the PPI group, training, explanation of trial methodology and terms, role clarification and development, good communication within and outside meetings, negotiation, conflict resolution, feedback on PPI contributors’ suggestions, and support and involvement from the Chief Investigator and other researchers. Material elements included having a suitable place to meet and adequate funding. All these factors support successful PPI [7, 10, 14, 15, 20, 21, 24].

Our PPI process was broadly consistent with previous recommendations about PPI implementation [10, 24], although only the INVOLVE guidelines were consulted at the trial outset. Our appraisal covers many of the questions raised in the PiiAF framework [17] and maps well to the standard operating procedure (SOP) developed by Evans et al. [20] in timeframe, planning, resourcing, PPI co-ordinator role, support from the Chief Investigator and involvement of the PPI group at all stages of the trial. Our experience also aligns with the findings of the RAPPORT study [7], which identifies six conditions most influential in establishing effective PPI. Four of these were evident in our study: a research team positive about PPI, a key individual to co-ordinate, ensuring diversity, and relationships that are established and maintained over time. The other two conditions, a shared understanding of moral and methodological purposes of PPI and proactive and systematic evaluation of PPI were at least partially met in our work.

In line with recommendations, we have reported on all aspects of the PPI process we undertook, largely complying with the reporting recommendations in GRIPP2 [12]. The GRIPP2 short form was insufficient for our purposes but our report does not entirely comply with the GRIPP2 longform either, since it was a retrospective appraisal and was not conceived as a research study of PPI in its own right. Therefore, we have not explicitly used either, although much of what we report maps to those checklists.

Our findings echo others’ experience that building good relationships can be challenging, requiring flexibility, clarity of expectations, and skilful facilitation [10, 25]. The incidence of significant conflict that led to one PPI contributor leaving the group highlighted the importance of clarifying needs and expectations at the outset. If this had been done, a compromise might have been negotiated that reconciled the different requirements individuals had to be able to participate.

Initially, the PPI co-ordinator felt there was an academic divide, with researchers sometimes seeming to defend against the PPI contributors’ comments, although this was a perception not necessarily shared by the PPI contributors. Tension can occur over who has control of the research [5, 25] and in this case may have resulted from a perception that PPI contributors wanted more influence over the research than researchers felt they could give. Later, slight tension arose because some members of the PPI group were interested in the impact of the intervention on individuals’ behaviour, whereas the trial design only allowed for generalisable results. Some members of the group would have liked more say in which outcomes were measured, and were frustrated in this by not being involved until the trial was fully designed. However, they did significantly contribute to development of a measure of treatment burden that was used in the trial. Ultimately, contributors’ wish to know more about intervention impact on individual patients was largely satisfied by sharing and inviting comment on anonymised data from the process evaluation. These qualitative data explored participants’ perceptions of the intervention.

Researchers had their own perception of what they wanted from the PPI group, which did not necessarily coincide with PPI contributors’ interests [18]. Another possible factor was that PPI contributors had divergent expectations and motivations that differed depending partly on the amount of PPI experience they had [14, 15, 24, 25]. However, the PPI co-ordinator perceived that as the relationships between the researchers and the PPI contributors evolved, mutual trust deepened, which was evident in increased openness in communication. Researchers seemed to relax and perceive greater potential for the PPI group to contribute to the trial. PPI contributors seemed to gain confidence and freedom to challenge which led to an enhanced sense of making a difference and a strong sense of ownership towards the trial [14, 15, 24].

Ultimately, both PPI contributors and researchers experienced personal benefits and satisfaction from their involvement and felt that challenge and difference produced constructive outcomes. PPI contributors engaged well with the research process and found the technical, clinical and procedural aspects of it interesting. The Chief Investigator, although already supportive of PPI, had not previously experienced such a high level of engagement, perhaps leading to revised expectations of PPI in future research [5]. Although there were some tensions, the expectations of researchers and PPI contributors regarding the research were sufficiently aligned to avoid the situation of ‘colliding worlds’ [5, 25]. The research team’s regular feedback on the impact of PPI contributors’ input was noted and valued, helping to maintain motivation and engagement [3, 15, 21].

Since the relationships that develop between researchers and PPI contributors are pivotal to the success of PPI [13, 24], the quality of the relationships may perhaps be used as an additional indicator of success, alongside the impact on the trial and those taking part. In line with other research, one PPI contributor suggested that the designated coordinator was key to constructive interaction [10, 24]. The co-ordinator should be experienced in the potential challenges of PPI, including power and control issues, and the consequences of PPI contributors lacking knowledge of research processes, terminology, and ethical constraints. In addition, the PPI co-ordinator should be sufficiently aware of the needs of the PPI group itself.

Despite some challenges, the diversity of PIP-CaRe group members was clearly perceived to be a strength in our study, because of their range of experience. This included prior research experience as there were both research-naïve and experienced PPI contributors in the group [10, 26]. Although the research-naïve contributors sometimes required lengthy explanations and made suggestions incompatible with research design and ethics constraints, they also asked thought-provoking questions. They used their own experience to provide a very real patient perspective, reminding researchers of the potential real-life impact of the research [15]. Experienced contributors, with greater understanding of research, seemed more confident to comment about and challenge the research design. These observations are reflected in the debate about whether previous PPI experience is desirable [10, 26] and linked to the question about the extent to which PPI contributors should be representative of the population on which the research is being carried out [4]. It is usually not feasible to achieve representativeness in PPI recruitment which relies on people volunteering [4]. Some may feel this is a flaw in PPI work, as did one researcher in our study with respect to literacy, but contributors may need to possess different characteristics from research partipicants, such as a certain level of literacy so that they can contribute. However, PPI contributors and research participants do need to share some characteristics, principally personal experience of the condition being researched.

Another strength of this study is that we have included diverse evidence of impact. The activity log provides evidence for impact on the trial, and the researchers’ and PPI contributors’ perspectives demonstrate impact on the experiential knowledge of both; knowledge that is gained through the interaction of individuals [13, 27]. However, whether the impacts on the trial translated into a difference in trial outcomes by influencing intervention implementation is not possible to determine. The group strongly supported early involvement and felt they might have had more impact if they could have contributed more to the actual research design including outcome measures, which echoes recommendations made by others [14, 15]. Although two PPI contributors sat on the trial steering committee and advisory group, the sense of co-ownership of the research and impact might have been strengthened if there had been a co-applicant from the target patient group involved from the very beginning of the trial design.

## Conclusion

Our PPI was wide-ranging, contributed to every stage of the trial and demonstrably had impact on trial processes. Both researchers and PPI contributors had a positive experience which will influence future PPI involvement. From this, we conclude that the PPI was successful. In fully reporting the PPI in the 3D study we have contributed to the evidence base regarding both ‘What difference does PPI make?’ and ‘What’s the best way to do it?’. This detailed study report may thereby help others to maximise their PPI group potential to contribute to and co-own the research and provides a useful addition to the published evidence of PPI in research.

## Appendix 1

### PPI input log

Meetings every 3 months attended by average 10 people each time.

Also had training delivered by CM in Dec 2014.

**Table:**

Timing	Contribution
During development of grant application	Confirmed relevance of intervention to their concerns. Strengthened emphasis on co-ordination of care
Dec 2013	Agreed on acceptable terms to be used: ‘Several long lasting health problems’ instead of co-morbid or multimorbidity ‘Patient’ and ‘intervention’ were acceptable ‘New approach’ also acceptable so long as not abbreviated to NA
Jan/March 2014	Insisted that patients lacking capacity to consent and carers should also be included in study.
March 2014	Voted on study logo design and strapline for trial
March 2014	Highlighted the importance of confidentiality of patient health data, particularly in the use of an external software company.
June–Sept 2014	Advised on formatting and wording of patient questionnaire and instigated changes to list of conditions and some wording. Additional introductory sentences were added on the front page of the questionnaire to express appreciation of participants’ contribution in completing it. A comments box was deemed necessary.
June 2014	Advised on formatting (1 column instead of multiple newpaper style columns) and wording of patient information sheet and invitation letter. Emphasised need for large fonts sizes and alternative formats for those with impaired vision.
June 2014	Advised on how to help the process of booking longer appointments. Suggested words or scripts for receptionist. Members volunteered to help role-play such scripts. Advised on wording of the 3D card to state ‘My usual GP is:…’ instead of ‘Your usual GP is…’
July–Aug 2014	9 members interviewed for development of new treatment burden questionnaire. 3 Carers were also interviewed to help the development of a treatment burden for carers questionnaire. Helped test and validate resultant questionnaires.
Sept 2014	Drew attention to potential concerns that participants might have about questionnaire results being made known to GP practices
Sept 2014	Further feedback on patient questionnaires: Suggested including the length of time required to complete questionnaire Highlighting where questions asked about their GP practice, that answers were confidential and would not affect the care they receive. Suggested other examples of social activities Queried the use of the word ‘Chronic’ – changed to ‘Long term’. Ways to improve formatting to help visually impaired eg spacing of boxes and shading to break up sections.
Sept 2014	5 members took part in focus group about usual care
6 Oct 2014	2 members attended Trial Steering Committee
Dec 2014	Helped with test-retest reliability of treatment burden questionnaire
5 Jan 2015	1 PPI representative attended the study Advisory group meeting
Mar 2015	Advised on revised patient information documents following pilot study. Group felt strongly that patients who did not want to take part must be given a way to say so to prevent further contact otherwise it may be seen as harassment. This suggested need for active decline if removing the decline form Suggested including a statement that patients were involved the design of the trial, to try to improve recruitment rates.
Mar 2015	1 member volunteered to help with analysis of qualitative patient data
23 March 2015	1 PPI representative attended the Trial Steering Committee
June 2015	PPI group had been asked general questions about how their conditions affect their life, what is difficult, what would help etc. Quotes were extracted to highlight the problems patients faced, to be used in the practice training sessions
June 2015	One PPI member volunteered to be video recorded in a mock consultation of a 3D nurse and GP review. This had been at the suggestion of the PPI representative on the TSC, and agreed amongst trainers and practice staff would be a useful resource.
Aug2015	1 member joined the CAPC steering group for PPI&E
29 Sept 2015	2 PPI representatives attended the Trial Steering Committee meeting
Oct 2015	Group provided suggestions about what CM should be asking in the interviews and focus groups with patients.
Oct 2015	Group also gave suggestions about what to look for in observing consultation reviews as evidence of patient-centredness. Eg. appropriate eye contact, opening and closing of review, open or closed questions, did their approach adapt to different patients?
22 Oct 2015	2 PPI representatives attended the study Advisory group meeting
Dec 2015	Group reviewed a Patient letter explaining the change in follow-up questionnaire timing (from 6 and 12 months, to 9 and 15 months). Wording clarified prior to submission as part of ethics amendment 10.
Feb 2016	The group reviewed the first patient newsletter. Those with visual impairments stressed the importance of text size and also where the contrast of text on coloured boxes made it difficult to read. Yellow text on dark blue background was preferred.
Feb 2016	Group reviewed the study website. Did not like the use of the word ‘managed’ and suggested replacing the main image with a photo of a female doctor with patient. Also suggested changes to headings, clarifying some words and definitions, highlighting where information needed updating, where navigation links were broken and PPI pages needed to be made more prominent.
Feb 2016	Total of 6 members took part in 2 focus groups to discuss opinions and experiences of medication reviews. This was to help shape a new sub-project about pharmacy reviews (led by PD).
April 2016	5 members offered to help with a goal setting project proposal led by CM.
May 2016	Group posed for photos to be used on the PPI pages of the study website Further feedback on study website. Suggested links to other helpful or relevant websites (listed as Other Resources).
May 2016	Identified 2 members to assisting with analysis (checking coding and developing themes) of qualitative patient data
Sept 2016	5 members were video recorded having a discussion about the importance of the 3D study and why they got involved. Extract to be made publically available on the study website.
26 Sept 2016	1 member attended the Trial Steering Committee
Oct 2016	Group feedback on audio excerpts from GP consultations. Opinions and comments used to corroborate examples of good and poor patient centred care.
Oct 2016	Review of second patient newsletter. Photo from the group discussion (Sept 2016) used on the newsletter.
Jan 2017	Group discussion of patient focus group transcripts and patient case study to explore views of experiences and help to validate interpretation of qualitative data.
Jan 2017	Discussion of study dissemination plan. Suggested local and national organisations, charities and media that could be approached to disseminate study results.
Jan – March 2017	Several members expressed an interest in writing a research paper about their contribution of being part of a PPI group involved in research. 7 members provided personal views to key questions. 2 members collated and wrote a draft of emerging themes.
April 2017	Fed back on draft of paper about PPI experiences. Discussions of different motivations and acknowledging that patients, researchers and health care professionals all have different perspectives. Expressed an interest to submit an abstract to the Involve conference.
April 2017	Provided suggestions on what information would be of interest to patients at the end of the study (for study patient newsletter).
28th April 2017	Abstract submitted for INVOLVE conference
May 2017	Abstract accepted for poster presentation at INVOLVE conference
June 2017	Results section of PPI in research paper written. Starting to develop discussion section of paper.
26 June 2017	Fed back on CM’s HSRUK conference presentation on the effect of the 3D template on patient centredness.

## Abbreviations

NIHR: National Institute of Health Research
PIP-CaRe: Patients In Primary Care Research
PPI: Patient and Public Involvement
RCT: Randomised Controlled Trial
